# Neurons in the primate dorsal striatum signal the uncertainty of object–reward associations

**DOI:** 10.1038/ncomms12735

**Published:** 2016-09-14

**Authors:** J. Kael White, Ilya E. Monosov

**Affiliations:** 1 Department of Neuroscience, Washington University School of Medicine, 660 S. Euclid Avenue, St Louis, Missouri 63110, USA

## Abstract

To learn, obtain reward and survive, humans and other animals must monitor, approach and act on objects that are associated with variable or unknown rewards. However, the neuronal mechanisms that mediate behaviours aimed at uncertain objects are poorly understood. Here we demonstrate that a set of neurons in an internal-capsule bordering regions of the primate dorsal striatum, within the putamen and caudate nucleus, signal the uncertainty of object–reward associations. Their uncertainty responses depend on the presence of objects associated with reward uncertainty and evolve rapidly as monkeys learn novel object–reward associations. Therefore, beyond its established role in mediating actions aimed at known or certain rewards, the dorsal striatum also participates in behaviours aimed at reward-uncertain objects.

## Abstract (web summary)

The dorsal striatum (DS) is a brain region that is thought to aim actions at certain or known rewards. Here, the authors show that an internal-capsule bordering region of the primate DS signals the uncertainty of object-reward associations, suggesting a novel role for the DS in behavior under uncertainty.

## Graphical abstract

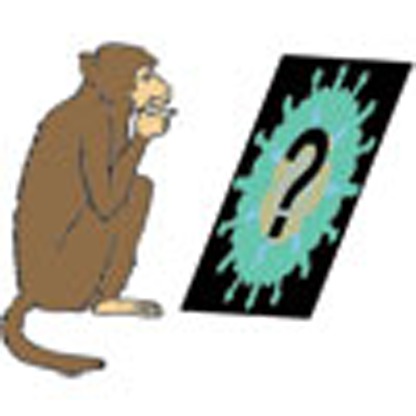

To survive, humans and other animals must act on objects that have been previously associated with certain or reliable rewards^1^,^2^,^3^. However, learning, foraging and decision-making also require animals to monitor, approach and act on objects associated with variable or unknown rewards^4^,^5^,^6^,^7^, even when the mean reward value of such uncertain objects is lower than that of other objects^8^,^9^,^10^. To date, the mechanisms that direct behaviour towards uncertain objects are not well understood.

Expected (or certain) reward-driven behaviours are in part dependent on the caudate–putamen complex^11^,^12^,^13^, also called the dorsal striatum (DS). In primates, the caudate nucleus in particular has recently been shown to contain multiple mechanisms for directing gaze at objects associated with high reward values^14^,^15^,^16^,^17^. Here we asked if the primate DS also contains a mechanism to support behaviour aimed at objects associated with outcome uncertainty.

Our experiments showed that a subset of neurons, mostly in the internal-capsule bordering regions of the DS (icbDS), was preferentially activated by visual objects associated with reward-uncertain outcomes. Furthermore the icbDS reward-uncertainty responses depended on the presence of visual objects associated with reward uncertainty because they were mostly ablated when the object was removed before the uncertain outcome was delivered. Finally, during object–reward associative learning, icbDS neurons' uncertainty responses evolved rapidly as monkeys learned novel object–reward associations. These uncertainty responses identified object associations that were uncertain either due to the subjects' lack of knowledge or due to known uncertainty (also called risk^18^,^19^).

Our experiments suggest that uncertainty-sensitive neurons in the primate DS may play important roles in object-based behaviours under uncertainty.

## Results

### DS neurons selectively signal reward uncertainty

To test if the primate DS contains neurons that are preferentially activated by visual objects associated with reward-uncertain outcomes, we recorded 141 single neurons from DS while two monkeys (B, *n*=103 neurons; W, *n*=38 neurons) participated in a behavioural procedure that was composed of two distinct blocks: a reward-probability block, in which three visual conditioned stimuli (CSs) predicted a 0.25 ml juice reward with 100, 50 and 0% chance; and a reward-amount block, in which three CSs predicted 0.25, 0.125 and 0 ml of juice (experiment 1). For each block, we used two fractal sets that could appear in one of three spatial locations. Monkeys' knowledge of the task was tested with interleaved choice trials (Methods), and neuronal recordings did not begin until the monkeys chose the CSs associated with higher expected value over CSs associated with lower expected value >90% of the time (Supplementary Fig. 1).

Uncertainty-sensitive neurons were defined as those that varied their responses across the task CSs (Kruskal–Wallis test; *P*<0.01) and displayed significantly stronger responses to the 50% CS than to both 100 and 0% reward CSs or weaker responses to 50% CS than to both 100 and 0% reward CSs (two-tailed rank-sum tests; *P*<0.01). We found that 45/141 neurons, mostly in the internal-capsule bordering regions of the striatum, were selectively activated by reward uncertainty (*n*=19 in monkey W; *n*=26 in monkey B). 0/141 neurons was selectively suppressed by uncertainty.

**Figure 1:**
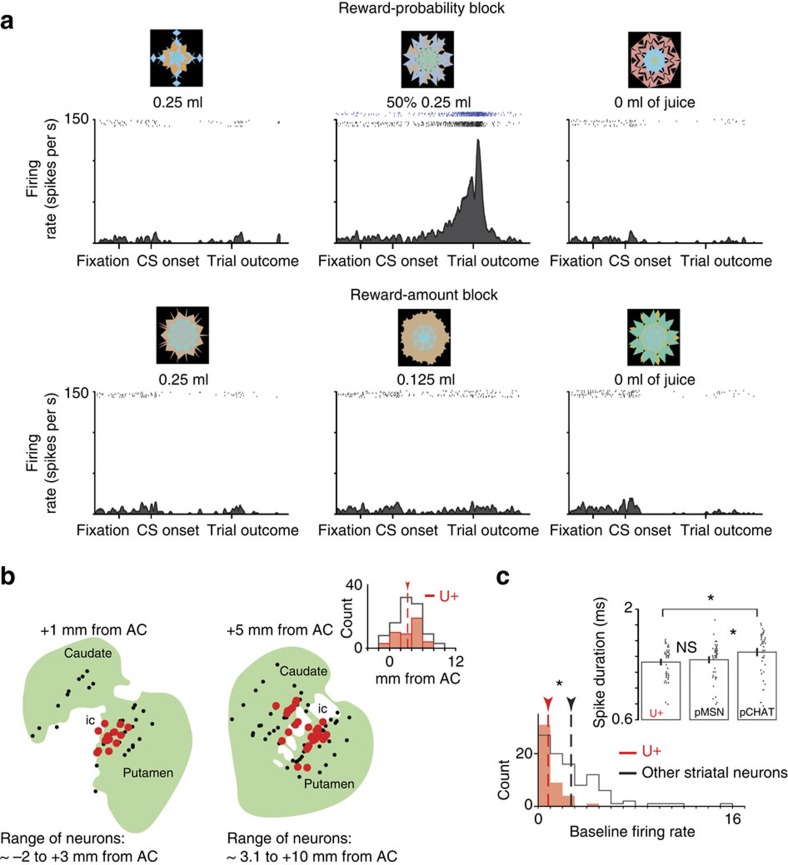
Selective reward-uncertainty responses in the DS. (**a**) Responses of a single uncertainty selective (U+) neuron in the internal-capsule bordering region of the striatum to the presentation of six fractal objects (shown above rasters) associated with certain and uncertain predictions of juice reward. Dark blue raster plots indicate the activity in 50% CS trials in which reward was omitted. (**b**) Estimated locations of 45 U+ neurons (red dots) in the internal-capsule bordering striatum shown on two coronal slices. Ranges of the neurons on each slice and the distance of each slice from the centre of the anterior commissure (AC) are indicated. Black dots indicate other recorded neurons. Inset is the histogram of recording locations along the anterior–posterior axis. U+ neurons (red) were most often found anterior to the AC. (**c**) Histogram of baseline firing rates of recorded neurons. Inset shows spike durations (trough-to-trough) for all U+ neurons (left), non-uncertainty-selective putative medium spiny neurons (pMSN; neurons with a baseline firing rate of <3 spikes per s), and non-uncertainty-selective putative cholinergic interneurons (pCHAT) neurons (neurons with a baseline firing rate <3 spikes per s). Error bars indicate standard errors. Single neuron data points are shown as scatters. Asterisks indicate significant differences (Wilcoxon rank-sum test; *P*<0.05).

An example uncertainty-sensitive (U+) neuron's CS responses are shown in Fig. 1a. Its activity increased following the presentation of the CS that predicted 0.25 ml of juice reward with 50% chance until the uncertain outcome was delivered and the uncertainty was resolved. This example neuron did not strongly respond to other CS objects or task events.

The location of all recorded U+ neurons is shown in Fig. 1b. U+ neurons were most often found within the anterior–dorsal putamen and caudate nucleus regions that bordered the internal capsule (Supplementary Figs 2–4), prominently in the anterior putamen. We refer to this brain area as the icbDS. The low baseline discharge rate of U+ neurons (mostly <1 spikes per s; Fig. 1c) suggests that they are medium spiny neurons^12^,^15^,^20^,^21^,^22^—the chief output neurons of the striatum.

**Figure 2:**
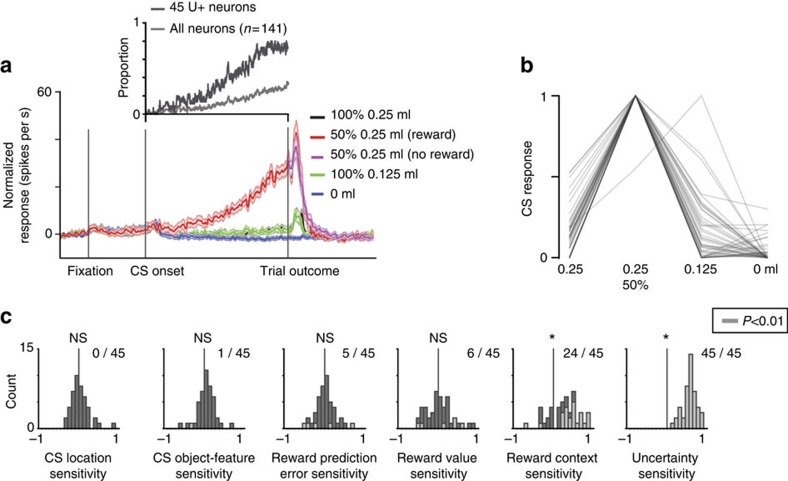
Population activity of U+ neurons. (**a**) Average responses of 45 U+ neurons to different reward predictions in the reward-probability and reward-amount procedure. Shaded region represents standard error. The inset shows proportion of neurons (of 45 U+ neurons and of all 141 striatal neurons) displaying uncertainty selectivity during the CS epoch in time. (**b**) CS responses of 45 U+ neurons for different reward predictions in the reward-probability and reward-amount procedure (normalized to the maximum CS response; from 0 to 1). In all, 44/45 neurons had the highest response for the 50% CS. (**c**) Sensitivity indices (Methods) for 45 striatal uncertainty-selective neurons for different behavioural/task variables. Asterisk above the histogram indicates significant deviation from 0 (*P*<0.01; sign-rank test). Significant individual neuron indices (*P*<0.01; Wilcoxon rank-sum test) are grey. The number of significant indices is indicated near the histogram.

All U+ neurons exhibited roughly similar responses (Fig. 2a,b). On average, they were strongly activated by the presentation of the CS that predicted 0.25 ml of juice reward with 50% chance. This activation was most often a ramp-like increase in activity, which continued until the uncertain outcome was delivered and the uncertainty was resolved (Fig. 2a). Amongst single neurons, 44/45 U+ neurons' responded more strongly to the CS object associated with 50% 025 ml of juice than to the CS object associated with 0.125 ml of juice (Fig. 2b) even though these CSs were associated with the same expected reward value.

Further neuron-by-neuron analyses revealed that amongst the task features of experiment 1, U+ neurons were consistently sensitive to reward uncertainty and to reward context (that is, difference between trials in which reward was possible versus trials in which rewards would not be delivered). This is shown in Fig. 2c and in Supplementary Fig. 5 for icbDS U+ neurons in caudate and putamen, separately. Most single U+ neurons did not encode information about expected values (defined as the difference between responses to objects associated with 0.25 and 0.125 ml of juice), spatial- and object-feature parameters (Fig. 2c), or aversive outcomes (Supplementary Fig. 6). However, 24/45 U+ neurons discriminated reward-associated CSs from CSs associated with no outcome delivery (Fig. 2c, this reward-related enhancement can also be observed in the average activity in Fig. 2a). Also, on average, U+ neurons responded to the delivery of expected/certain rewards with a weak but consistent phasic excitation (Fig. 2a; *P*<0.05; sign-rank test). The observations in Fig. 2 indicate that while U+ were preferentially dedicated to signalling reward uncertainty, they were also sensitive to reward context (or expectation) and reward delivery.

While U+ neurons did not encode the locations of CS objects, thus far, it was unknown if they respond before or during saccades aimed at reward uncertain objects. To assess this further, we studied the dynamics of U+ uncertainty selectivity during choice trials. We found that, on average, U+ uncertainty selectivity emerged after the monkeys fixated the object associated with reward uncertainty (Supplementary Fig. 7). Therefore, U+ neurons did not trigger saccades aimed at reward-uncertain objects.

Overall, the results of experiment 1 showed that the icbDS contains a subpopulation of neurons with striking sensitivity to objects associated with reward uncertainty. However, several important questions about these neurons remained unclear. First, are they sensitive to the level of uncertainty in a graded manner^7^,^23^? Second, do U+ neurons signal internal states related to the expectation of reward or are their uncertainty responses dependent on external cues or objects? Third, can U+ neurons support object learning under uncertainty? To answer these important questions, we selectively recorded from U+ neurons in the icbDS in experiments 2–4.

### icbDS neurons are sensitive to the level of reward uncertainty

To test if U+ neurons were sensitive to the level of reward uncertainty, in experiment 2, we recorded 20 U+ neurons (14 in monkey B and 6 in monkey W) in a behavioural procedure in which monkeys experienced a reward-probability block that contained five objects associated with five probabilistic reward predictions (0, 25, 50, 75 and 100% of 0.25 ml of juice), and a reward-amount block that contained five objects associated with 100% reward predictions of varying reward amounts (0.25, 0.1875, 0.125, 0.065 and 0 ml)^7^,^23^. The expected values of the five CSs in the probability block matched the expected value of the five CSs in the amount block.

Reward-uncertainty neurons in icbDS were identified during online screening as neurons that responded to any of the uncertain conditioned stimuli (25, 50 or 75% reward). The same preselection criteria were used in subsequent experiments in this study and in our previous reports^7^,^23^.

**Figure 3:**
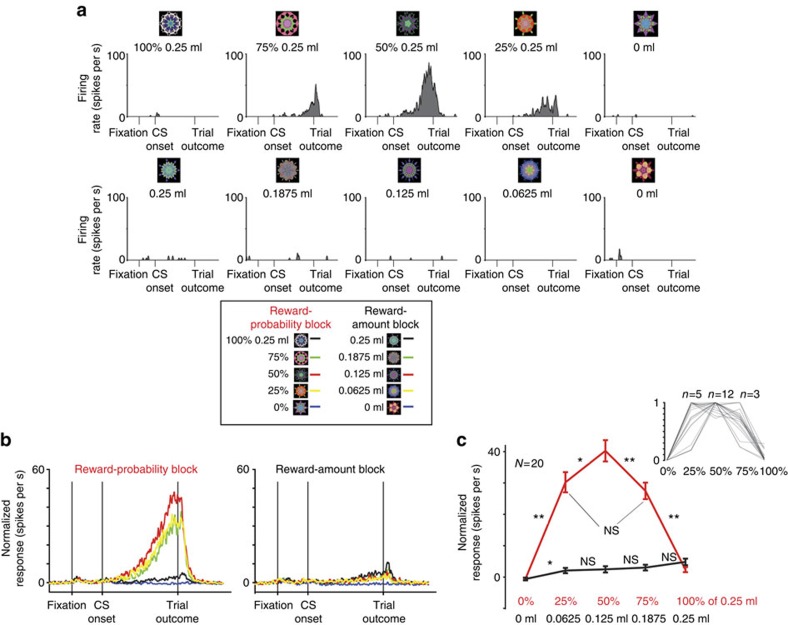
Striatal U+ neurons are sensitive to the level of reward uncertainty. (**a**) Responses of a single uncertainty selective (U+) neuron to the presentation of 10 fractal objects associated with certain and uncertain predictions of juice reward. (**b**) Average responses of 20 U+ neurons in the reward-probability block (left) and reward amount block (right). (**c**) Average normalized responses of 20 U+ neurons for probability (red) and amount (black) CSs. Asterisks indicate differences between CSs (**P<0.01; *P<0.025; paired sign-rank test). The inset shows the single neuron's CS responses for different reward predictions in the reward-probability block (normalized to the maximum CS response; from 0 to 1). Numbers above the inset indicate the number of cells that exhibited the greatest response for 25, 50 or 75% CSs; 60% of the neurons exhibited greatest response for 50% reward CS.

An example U+ neuron's responses to the 10 CS objects are shown in Fig. 3a. It responded most strongly to the presentation of the 50% CS object, and less strongly to the presentation of the 25 and 75% CS objects. Moreover, it did no respond to the presentation of objects associated with certain reward predictions (0 and 100% reward CS objects and CS objects in the reward-amount block). A similar result can be observed across the population of U+ neurons (Fig. 3b,c). U+ neurons' average response was strongest for the presentation of the 50% CS object. Their responses were weaker for 25 and 75% reward-associated CS objects. On average, there was no significant difference between their responses to the 25% versus 75% CS objects, which have the same level of uncertainty but different expected values. Furthermore, as in experiment 1, during the reward-amount block, the neurons discriminated objects associated with rewards from objects associated with no reward (Fig. 3c, black trace). In sum, experiment 2 showed that U+ neurons were sensitive to the levels of reward uncertainty.

### icbDS uncertainty responses are object-dependent

The results of experiments 1 and 2 are consistent with two scenarios. First, U+ responses may signal internal states related to reward expectation, particularly with the expectation of uncertain rewards. A second scenario is that U+ responses may signal the uncertainty of the object–reward associations, rather than the internal state associated with reward uncertainty. To distinguish between these alternatives, monkeys were presented with four CSs (experiment 3). Two distinct CSs were associated with 100 and 50% chances of reward and were kept on the experimental presentation screen for 2.5 s, until the time of the trial outcome (same trial structure as in Fig. 1a). Two other CSs were also associated with 100 and 50% chances of reward and were present on the screen for 1 s and outcomes were delivered in 1.5 s after the removal of the CSs (the 1.5 s period during which the CS is not present is referred to as a trace period). Therefore, for all CSs, reward was delivered 2.5 s after CS onset. Monkey performance indicated that they understood the procedure and were similarly motivated by trace and no-trace 50% reward predictions (Supplementary Fig. 8).

**Figure 4:**
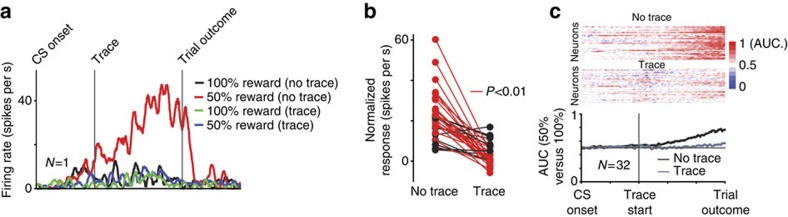
Striatal U+ neurons' responses are object-dependent. (**a**) Responses of a single U+ neuron to 100 and 50% reward predictions without a trace period (CS objects remained until the outcome) (black and red), and with a trace period (CS objects disappeared after 1 s) (green and blue). (**b**) In all, 22/32 neurons displayed significant differences in reward-uncertainty responses across the no-trace and trace conditions (red; rank-sum test; *P*<0.01; 26/32 were significant with a 0.05 threshold). All significant changes were reductions of uncertainty responses. Here normalization was performed by subtracting 100% CS responses from 50% CS responses (for trace and no-trace conditions, separately). (**c**) Single neuron (insets above) and population reward-uncertainty discriminability was greatly diminished in the trace condition. AUC, area under receiver-operating characteristic curve.

We identified U+ neurons in icbDS and recorded their activity in this paradigm (*n*=32 neurons; 11 in monkey W and 21 in monkey B). An example U+ neuron is shown in Fig. 4a. This neuron robustly discriminated 50% reward-associated CS object (uncertain condition) from the 100% reward-associated CS object (*P*<0.01; rank-sum test). But surprisingly, the removal of the uncertain CS (trace condition) before the outcome was delivered completely abolished its uncertainty selectivity (Fig. 4a, green and blue traces). Similar results were found for most of the U+ neurons (Fig. 4b). The discriminability of striatal uncertainty signals was greatly diminished when the uncertain object was not present at the time of the outcome (Fig. 4c). Many U+ neurons' uncertainty signals were completely abolished (Fig. 4b,c). These results indicate that U+ neurons' reward-uncertainty responses are contingent on the presence of the uncertain object.

In the basal forebrain (particularly in its medial regions), some neurons also signal reward uncertainty with ramp-like responses^23^, however, additional experiments revealed that their uncertainty-selective signals persist during the same trace-conditioning procedure used to study U+ neurons (Supplementary Fig. 8). Consistent with this observation, other reward-related signals are preserved during trace conditioning in brain regions that are interconnected with the basal forebrain, such as in the dorsal raphe^24^ and in the amygdala^25^. These observations suggest that basal forebrain and related limbic structures signal values and uncertainty of internal states (perhaps somewhat independently of the external environment), whereas the U+ neurons in the basal ganglia signal reward uncertainty associated with objects.

### icbDS uncertainty responses are rapidly shaped by learning

**Figure 5:**
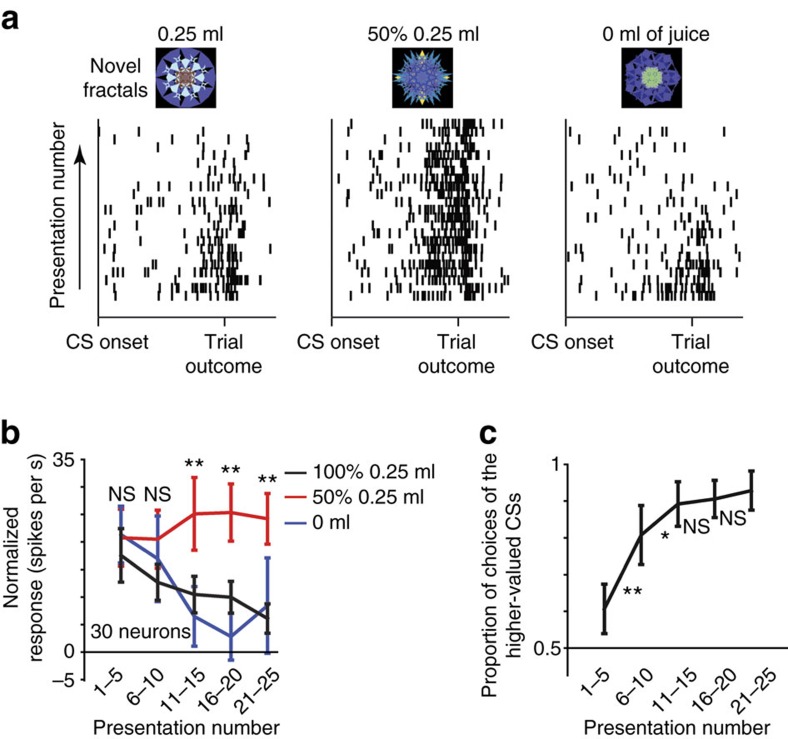
U+ responses are rapidly shaped by learning. (**a**) Single neuron's responses (shown as single trial rasters) to the presentation of three novel objects shown in the order of the monkey's experience (bottom to top). (**b**) Binned neuronal population response across learning (30 learning sessions, 30 neurons) shown separately for 100, 50 and 0% reward-associated novel objects. Asterisks indicate significant variance across the three conditions (*P*<0.01; Kruskal–Wallis test). Neuronal responses are shown separately for Pavlovian and choice trials in Supplementary Fig. 9. (**c**) Monkeys' choices during learning. Proportion of choices of the higher-valued fractal CS objects during randomly interleaved choice trials (binned like neuronal activity in **b**). **P<0.01, *P<0.05 (sign-rank test assessing difference between bins).

The data thus far prompted us to assess how U+ neuronal responses are shaped by the learning of novel object–reward associations (experiment 4). Thus, far we tested the responses of U+ neurons to reward uncertainty arising from knowledge about reward variability associated with 50% reward CSs (also called known-uncertainty or risk). But, if uncertain object–reward signals in the DS contribute to object learning, then U+ neurons should also signal uncertainty that is due to a lack of previous object–outcome associations (also called ambiguity)—an uncertainty that can be identified and resolved by learning. To test this, we recorded the activity of identified U+ neurons in a Pavlovian procedure in which three novel fractals were used as CSs associated with 100, 50 and 0% reward probabilities (*n*=30 neurons; 11 in monkey W and 19 in monkey B). One example U+ neuron is shown in Fig. 5a. At the start of learning, this neuron showed a strong increase in response to all the novel CSs. As the CSs were repeatedly experienced, the neuronal activity started to decrease for certain CSs (0 and 100%) and remained roughly the same for the reward-uncertain CS (50% reward prediction). The population of 30 U+ neurons shows a similar pattern (Fig. 5b and Supplementary Fig. 9). The neuronal responses to certain object–reward associations decreased as the monkeys learned (Fig. 5c). These results demonstrated that U+ neurons signal object–reward uncertainty of unknown or novel objects and that the DS uncertainty responses can be rapidly shaped by learning, even within a single experimental session.

## Discussion

In the caudate–putamen complex we found a population of neurons that signal uncertainty of object–reward associations. These U+ neurons were often found in the icbDS. Their uncertainty-selective responses depended on the presence of objects associated with reward uncertainty and evolved rapidly as monkeys learned novel object–reward associations.

What brain regions supply reward uncertainty signals to U+ neurons? Their average location in the striatum may provide a clue. U+ neurons were most often found within the anterior putamen and caudate regions that bordered the internal capsule (icbDS), prominently in the anterior putamen. icbDS receives inhibitory inputs from the ventral pallidum^26^, where some neurons are inhibited by reward uncertainty (Supplementary Fig. 10)^27^. Given the uncertainty-excitatory responses of many icbDS neurons (Fig. 2), we hypothesize that the inhibition of pallidal neurons by uncertainty may open a gate, so that U+ neurons can selectively respond to cortical inputs carrying sensory information about objects^28^,^29^ and about their reward value or uncertainty^30^. But precisely what cortical regions send uncertainty and other signals to U+ neurons remains to be assessed.

The task responses of striatal U+ neurons differentiated them from reward uncertainty-selective neurons in the anterodorsal septum and the medial basal forebrain. For example, during object learning, anterodorsal septal uncertainty-selective neurons responded preferentially to knowledge-based uncertainty (often called risk), after monkeys learned the uncertain stimulus–response association^7^. In contrast, during a similar object-learning task, U+ neurons responded strongly to novel stimuli, whose conditioned stimulus–unconditioned stimulus relationship was not yet learned (Fig. 5). Unlike U+ neurons, medial basal forebrain reward uncertainty-sensitive neurons slowly learned to discriminate between certain and uncertain reward-predicting objects^23^. This slow learning was not correlated with the fast time course of the monkeys' object–reward associative learning^23^. These data are consistent with the observation that there are no known connections from the medial basal forebrain or septum to the striatum and suggest that U+ neurons belong to a mostly distinct system for signalling uncertainty of objects that may be particularly well suited to contribute to object learning.

It is noteworthy that U+ neurons did not encode all types of uncertainty, or only uncertainty^7^,^19^,^31^. First, they did not respond to uncertainty about punishments. Whether there are neurons that signal uncertainty about all salient events (such as uncertainty about rewards and punishments) remains a mystery. Second, on average, they discriminated reward-associated CSs from reward-unassociated CSs (Fig. 2a,c). In fact, similar reward-related tonic activity shifts were observed in other neurons that encode reward uncertainty^7^,^23^. It remains to be tested whether they are due to context value (or relevance), or if they are due to uncertainty that could exist even during the expectation of ‘certain' rewards (for example, due to errors in the estimation of reward timing). Third, U+ neurons' uncertainty responses were abolished by the removal of the CS before the trial outcome (during trace conditioning). This suggests that striatal U+ neurons' responses depended on the presence of the uncertain CS object. This finding further differentiated striatal U+ neurons from uncertainty-enhanced neurons in the medial basal forebrain whose uncertainty selectivity persisted when the CS object was removed before the trial outcome (Supplementary Fig. 8).

Our study in monkeys and a previous human brain-imaging study^32^ suggest that icbDS is a prominent node for processing information about reward uncertainty. However, it remains possible that there are other striatal mechanisms for signalling uncertainty, and/or for integrating uncertainty with stimulus-feature information, movement kinematics and values^33^,^34^. Indeed, different areas of the primate striatum learn and signal values in distinct manners^11^,^14^,^16^,^17^,^33^,^34^,^35^,^36^,^37^ to support their different roles in action, decision-making, and learning and memory^11^,^14^,^15^,^17^,^29^,^33^,^34^,^36^,^38^,^39^,^40^. How uncertainty guides computations across different striatal subregions must therefore be an important direction of future studies.

Objects in the environment are important because they signal rewards or dangers, or because they represent an opportunity to learn and change one's state. In this study, we showed that the basal ganglia signals reward uncertainty of object–reward associations—a critical variable for monitoring and learning from objects. These results demonstrate a novel role for internal-capsule bordering putamen and caudate in controlling behaviours in uncertain contexts.

## Methods

### General procedures

Two adult male rhesus monkeys (*Macaca mulatta*) were used for the neurophysiology experiments in the DS (Monkeys B who is 6 years old; and Monkey W who is 5.25 years old). All procedures conformed to the Guide for the Care and Use of Laboratory Animals and were approved by the Washington University Institutional Animal Care and Use Committee. A plastic head holder and plastic recording chamber were fixed to the right side of the skull under general anaesthesia and sterile surgical conditions. The chambers were tilted laterally by 35° and aimed at the anterior portion of the striatum. After the monkeys recovered from surgery, they participated in the behavioural and neurophysiological experiments.

### Data acquisition

While the monkeys participated in the behavioural procedures we recorded single neurons in the right DS. The recording sites were determined with 1 mm-spacing grid system and with the aid of magnetic resonance images (3 T) obtained along the direction of the recording chamber. This magnetic resonance imaging-based estimation of neuron recording locations was aided by custom-built software (PyElectrode). Single-unit recording was performed using glass-coated electrodes (Alpha Omega). The electrode was inserted into the brain through a stainless-steel guide tube and advanced by an oil-driven micromanipulator (MO-97A, Narishige). Signal acquisition (including amplification and filtering) was performed using Alpha Omega 44 kHz SNR system. Action potential waveforms were identified online by multiple time-amplitude windows with an additional template-matching algorithm (Alpha-Omega). Neuronal recording was restricted to single neurons that were isolated online. Neuronal and behavioural analyses were conducted offline in Matlab (Mathworks, Natick, MA).

Eye position was obtained with an infrared video camera (Eyelink, SR Research). Behavioural events and visual stimuli were controlled by Matlab (Mathworks, Natick, MA) with Psychophysics Toolbox extensions. Juice, used as reward, was delivered with a solenoid delivery reward system (CRIST Instruments). Juice-related anticipatory licking during the CS epoch was measured and quantified using previously described methods^23^.

### Reward-probability and reward-amount procedure (experiment 1)

The reward-probability and reward-amount behavioural procedure consisted of two blocks, a reward-probability block and a reward-amount block (Fig. 1). In the reward-probability block, three visual fractal CSs were followed by a liquid reward (0.25 ml of juice) with 100, 50 and 0% chance, respectively. In the reward-amount block, three CSs were followed by a liquid reward of 0.25, 0.125 and 0 ml, respectively. Thus, the expected values of the three CSs matched between the probability and amount blocks. To control for neuronal object preference, we used two fractal sets (that is, for every CS there were two different fractals).

Each trial started with the presentation of a green trial-start cue at the centre. The monkeys had to maintain fixation on the trial-start cue for 1 s; then the trial-start cue disappeared and one of the three CSs was presented pseudo randomly. After 2.5 s, the CS disappeared, and juice (if scheduled for that trial) was delivered. The monkeys were not required to fixate on the CSs. In each trial, the CS could appear in three locations: 10° to the left or to the right of the trial-start cue, or in the centre. One block consisted of 18 trials with fixed proportions of trial types (each of the three CSs appears three times each block, 9/18 trials total).

In the remainder of the trials in each block (9/18), the monkeys chose amongst the task CSs. Each trial started with the presentation of a purple trial-start cue at the centre, and the monkeys had to fixate it for 0.5 s. After the monkey fixated on the trial start cue for 0.5 s, a choice array was presented consisting of two fractals used in the Pavlovian procedure (shown in Fig. 1a). The monkey had to continue to fixate until the trial start cue disappeared (0.5 s). Monkeys then made saccadic eye movements to their preferred reward-associated fractals and fixated them for 0.75 s to indicate their choices. Then, the unchosen stimulus disappeared, and the monkeys waited for 1 s to receive the scheduled outcome (associated with their chosen fractal).

The inter-trial intervals ranged from 3 to 6 s. Approximately one in five inter-trial intervals contained uncued events (chosen randomly). These could be either a juice reward alone (0.25 ml) or an ∼70 dB 0.15 s auditory white noise burst paired with a brief change in screen colour (same duration as the auditory stimulus).

Neuronal recordings did not begin until the monkeys chose the CSs associated with higher expected value over CSs associated with lower expected value >90% of the time. The monkeys' knowledge of the CSs was further confirmed when we measured the monkeys' licking behaviour. The magnitude of licking was correlated to the reward value of the fractals in the reward-probability block (*P*<0.001; Spearman's rank correlation) and the reward-amount blocks (*P*<0.001; Spearman's rank correlation).

### Five reward-probability and reward-amount procedure (experiment 2)

The reward-probability and reward-amount behavioural procedure consisted of two blocks, a reward-probability block and a reward-amount block. The trial structure was the same as in experiment 1. However, here the reward-probability block contained five objects associated with five probabilistic reward predictions (0, 25, 50, 75 and 100% of 0.25 ml of juice) and a reward-amount block that contained five objects associated with 100% reward predictions of varying reward amounts (0.25, 0.1875, 0.125, 0.065 and 0 ml)^7^,^23^. One block consisted of 20 trials with fixed proportions of trial types (each of the five CSs appears four times each block).

### Trace reward-probability procedure (experiment 3)

The temporal structure of this procedure was the same as in probability-amount procedure (experiment 1). The trace procedure contained four possible distinct CS fractals. The first two CSs were associated with 100% (CS 1) and 50% (CS 2) chance of 0.25 ml of juice. These CSs remained on the screen for 2.5 s and were followed by the scheduled reward outcome. (same as in experiment 1). The other two CSs were also associated with 100% (CS 3) and 50% (CS 4) chance of 0.25 ml of juice but were only presented for 1 s. This was followed by a 1.5 s trace period, during which the screen did not contain any stimulus. The trace period was followed by the scheduled reward outcome. Therefore, in both trace and non-trace conditions, monkeys experienced two types of reward predictions (certain and uncertain) and experienced outcome delivery in 2.5 s after the initial CS presentation.

### Object learning procedure (experiment 4)

Instead of using previously conditioned object fractals, monkeys were exposed to three novel CSs associated with 100, 50 and 0% chance of reward delivery. The task design and temporal structure of the trials were the same as in probability-amount procedure (experiment 1). However, the interleaved choice trials were choice trials amongst the three novel fractals.

### Appetitive–aversive procedure

The procedure consisted of two alternating blocks: appetitive and aversive^23^. In the appetitive block, three visual fractal CSs were followed by a liquid reward (0.4 ml of juice) with 100%, 50% and 0% chance, respectively. In the aversive block, three visual fractal CSs were followed by an air puff with 100%, 50% and 0% chance, respectively. Airpuff (∼35 psi) was delivered through a narrow tube placed 6–8 cm from the monkey's face. Temporal structure of the trials was the same as in other procedures, but here monkeys were not required to fixate the trial start cue. Each block consisted of 12 trials with fixed proportions of trial types (100%, four trials; 50%, four trials; 0%, four trials).

### Data processing and statistics

Spike-density functions were generated by convolving spike times with a Gaussian filter (*σ*=50 ms). To display single neurone examples (Figs 1a, 3a, and 4a) spike-density functions were generated by convolving spike times with a 100 ms Gaussian filter. A neuron was defined as uncertainty sensitive if its responses varied across the four possible reward predictions (100% 0.25, 50% 0.25, 100% 0.125 and 0 ml of juice) (Kruskal–Wallis test, *P*<0.01; analysis window: 100 ms after CS presentation until outcome) and if its response to the uncertain CS (50%) was significantly stronger or weaker than its responses to both 100 and 0% reward CSs (two-tailed rank-sum test; *P*<0.01). The same analysis window was used to study neuronal activity during the CS epoch in Fig. 2c.

To normalize task-event-related responses, we subtracted baseline activity (the last 500 ms of the inter-trial interval) from the activity during the task-event-related measurement epoch. All statistical tests were two-tailed. For comparisons between two task conditions for each neuron, we used a rank-sum test, unless otherwise noted. For comparisons between two task conditions across the population average we used a paired signed-rank test, unless otherwise noted. Statistical threshold throughout this study is *P*<0.01 unless otherwise noted.

To assess the sensitivity of individual uncertainty-selective striatal neurons to task-related variables in Experiment 1 (Fig. 2c), we obtained their response indices (difference between neuronal responses to two conditions divided by their sum). To assess CS spatial location sensitivity, we compared responses to the 50% CS when it was shown 10° to the right versus 10° to the left of centre. To assess object-feature sensitivity, we compared responses to two distinct 50% CS fractal objects. Reward-value sensitivity was assessed by comparing neuronal responses to 100% 0.25 ml CS versus 0.125 ml CS. Reward-context sensitivity was assessed by comparing CS activity in certain reward trials (100% 0.25 and 0.125 ml CS trials) versus no reward trials. Uncertainty sensitivity was assessed by comparing responses to 50% reward CSs with 100% reward CSs. Reward prediction error sensitivity was assessed by comparing reward versus no-reward responses after the 50% reward prediction (in the 250 ms window after the outcome). Neuronal responses during experiments 2–4 were measured in the last 500 ms before the trial outcome.

To calculate receiver-operating characteristic (ROC) that assessed neuronal discrimination of uncertainty, we compared spike-density functions of 100% reward CS trials and 50% reward CS trials. The analysis was structured so that receiver-operating characteristic area values >0.5 indicate that the activity in the 50% reward CS trials is greater than in the 100% reward CS trials values <0.5 indicate that the activity in the 100% reward CS trials is greater than in the 50% reward CS trials.

### Data availability

Data supporting the findings of this study are available within the article and its Supplementary Information Figures or from the authors on request.

## Additional information

**How to cite this article**: White, J. K. & Monosov I. E. Neurons in the primate dorsal striatum signal the uncertainty of object–reward associations. *Nat. Commun.* 7:12735 doi: 10.1038/ncomms12735 (2016).

## Supplementary Material

Supplementary Information Supplementary Figures 1-10
