# Supplementary material for: Neurons in the primate dorsal striatum signal the uncertainty of object–reward associations
Source: Nat Commun. 2016 Sep 14;7:12735. doi: 10.1038/ncomms12735 (PMC5027277; doi:10.1038/ncomms12735)
Supplement: Supplementary Information — Supplementary Figures 1-10 [file ncomms12735-s1.pdf]

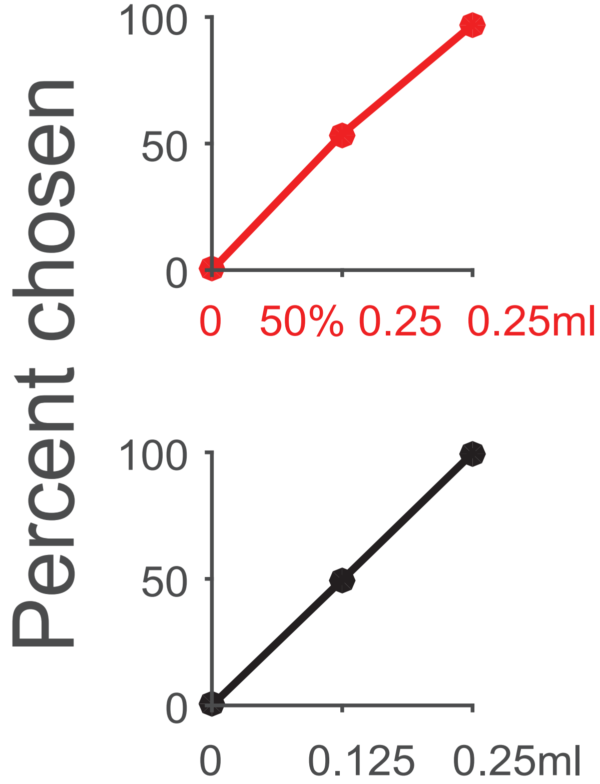

Supplementary Figure 1 – Monkeys' choice behaviour in Experiment 1

Choice percentage of a single reward-probability CS (x-axis) versus all the other reward-probability CSs (red). Choice percentage of a single reward-amount CS (x-axis) versus all the other reward-amount CSs (black). Data compiled from 5602 trials.

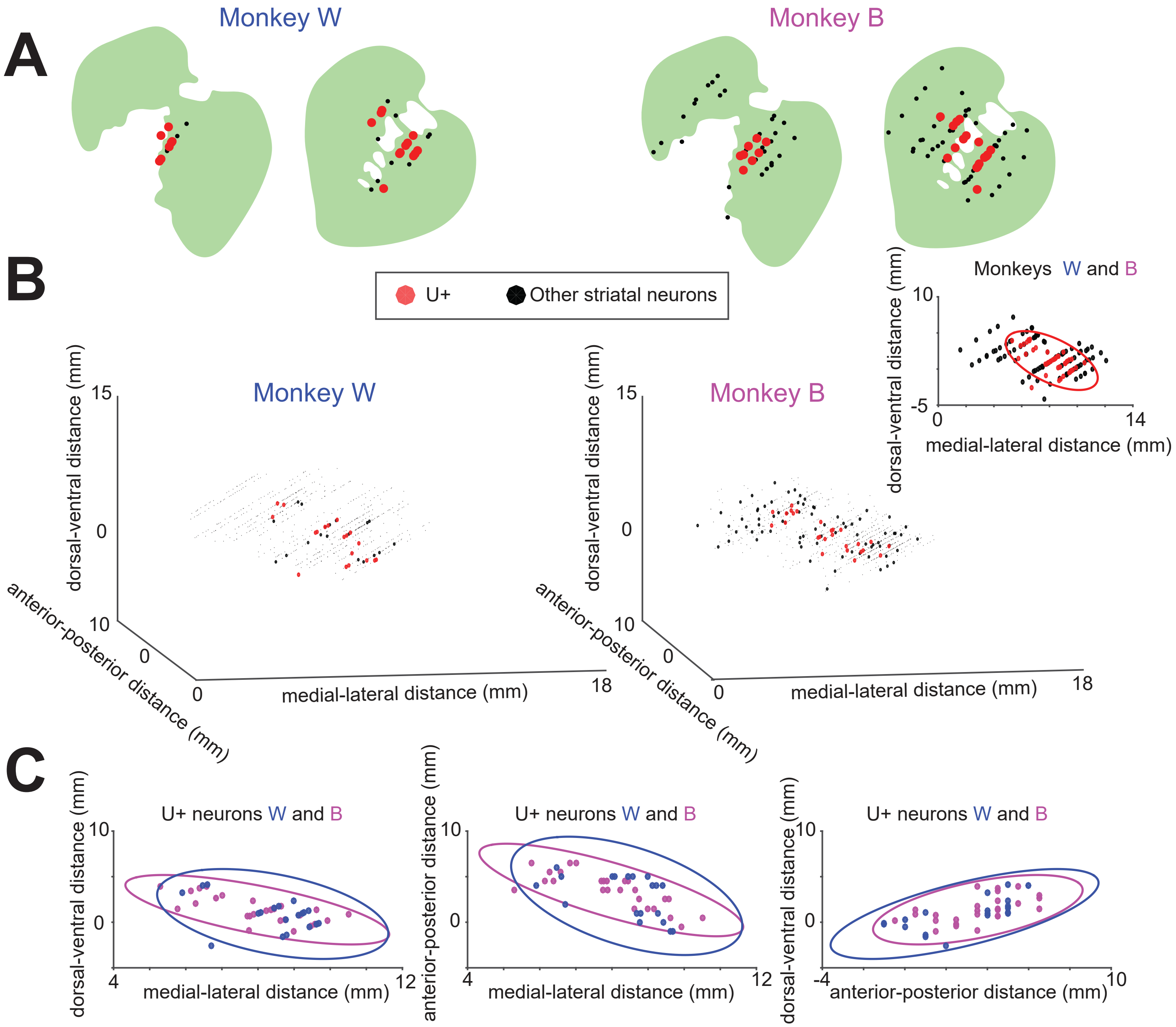

Supplementary Figure 2 – Supplementary information about the location of U+ neurons

(**A**) Estimated locations of U+ neurons (red dots) in the internal capsule bordering striatum shown on two coronal slices for each monkey. Formatting, anterior-posterior ranges of the neurons on each slice, and the distance of each slice from the center of the anterior commissure (AC) are as in Figure 1. (**B**) Three dimensional scatters of neuronal locations relative to the center-top of the AC. Red dots – U+, Black dots – other recorded striatal neurons, small black dots – neurons that were encountered (but not recorded) during experiments to map the extent of the caudate and putamen and to verify locations of the internal capsule and AC. Inset – locations of recorded neurons from two monkeys shown in two planes (medial-lateral versus dorsal-ventral) relative to the center-top of the AC. 95% confidence ellipse (red) around the U+ neurons includes 44 U+ neurons and 65 other recorded striatal neurons; 13 of those 65 neurons displayed significant correlations with the expected reward values of the CSs (correlations were assessed as relationship between the size of expected rewards in the reward amount block and neuronal activity during the CS epoch; Spearman's rank correlations; $p<0.05$; tested using a permutation test; 10,000 shuffles). (**C**) U+ neurons shown for two monkeys separately (magenta and blue) relative to the center-top of the AC. Each plot compares two different anatomical planes. 95% confidence ellipses of the two monkeys in all planes were highly overlapped.

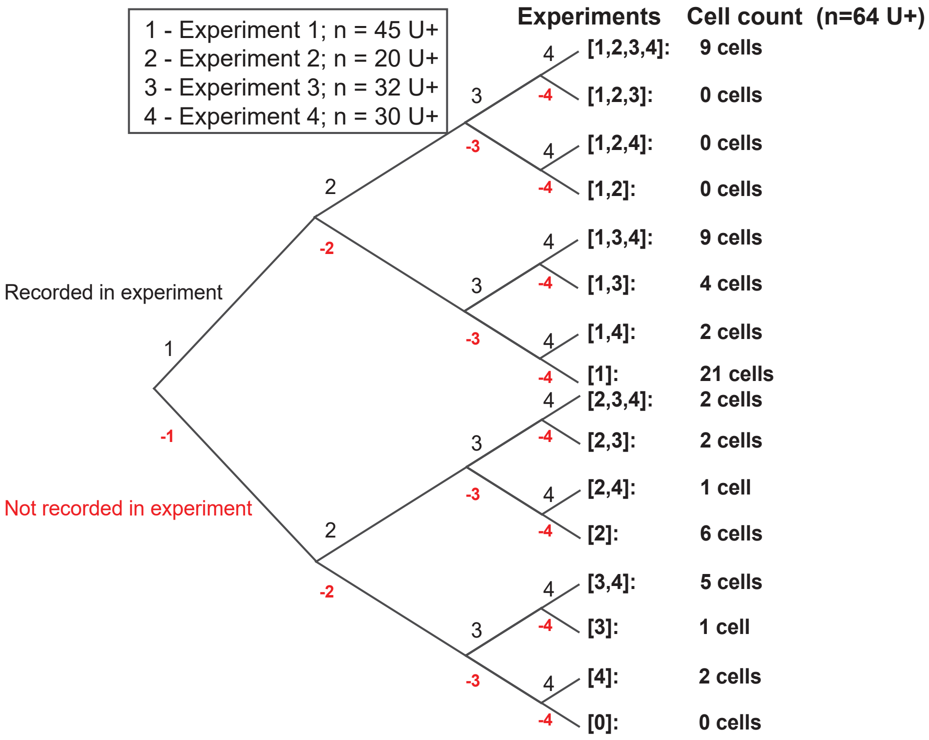

Supplementary Figure 3 – Supplementary information about neuronal recordings across tasks

124 electrode penetrations into the internal capsule bordering regions of the striatum yielded 64 U+ neurons. The classification tree shows how they were sampled across four experiments. The inset shows the number of neurons that were sampled for each experiment.

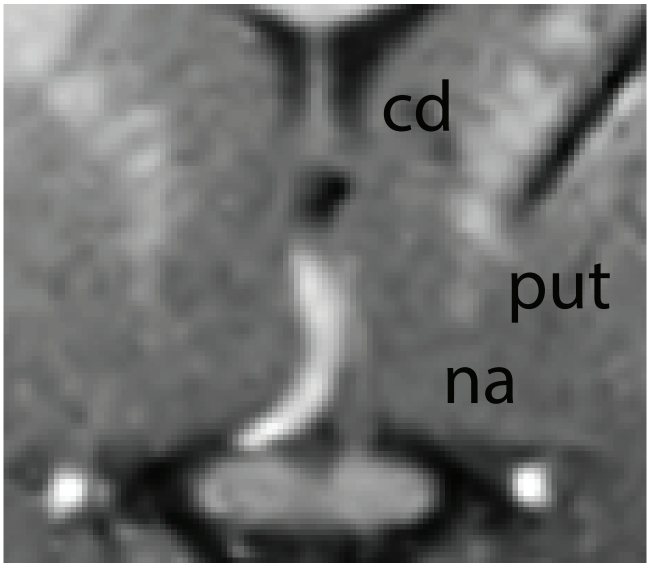

Supplementary Figure 4 – A coronal image confirming a recording location of a U+ neuron in internal capsule bordering striatum (icbDS)

Coronal T1 magnetic resonance (MR) image taken with a tungsten electrode at the location of an identified U+ neuron. The electrode's shadow is the black line whose tip is in icbDS. cd, caudate nucleus, na, nucleus accumbens; put, putamen.

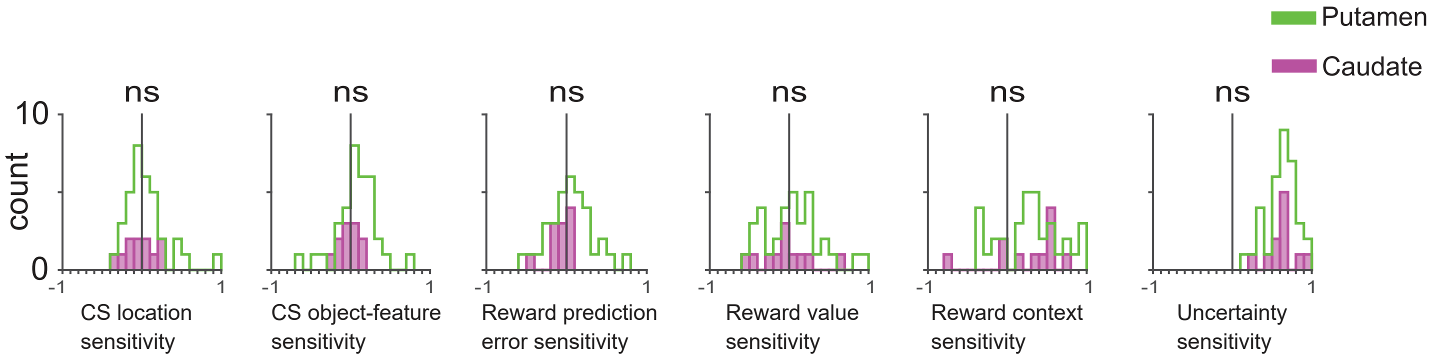

Supplementary Figure 5 – Sensitivity indices comparing U+ neurons in the putamen versus caudate nucleus

Sensitivity indices (same as in Figure 2) are shown separately for putamen and caudate U+ neurons. There were no significant differences between neuronal task-responses amongst populations of putamen and caudate U+ neurons (ns; Wilcoxon rank sum test).

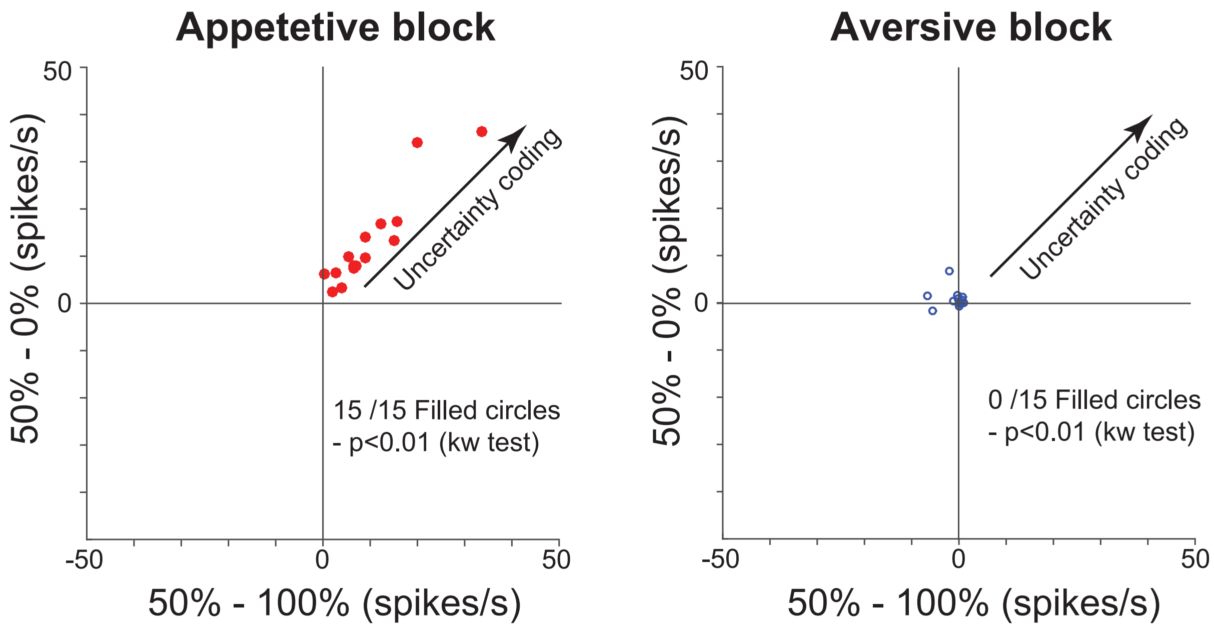

Supplementary Figure 6 – U+ neurons' responses in the Appetitive-Aversive procedure

For each neuron (indicated by a circle; n=15; Monkey B), we plotted the differences in response magnitude between 50% reward CS and 100% reward CS (x-axis) and between 50% CS and 0% reward CS responses (y-axis). The responses in the appetitive and aversive blocks are shown separately (left and right). Filled circles indicate neurons that displayed significant variability across the 3 reward (left) or punishment (right) predicting CSs (Kruskal-Wallis test; $p<0.01$)

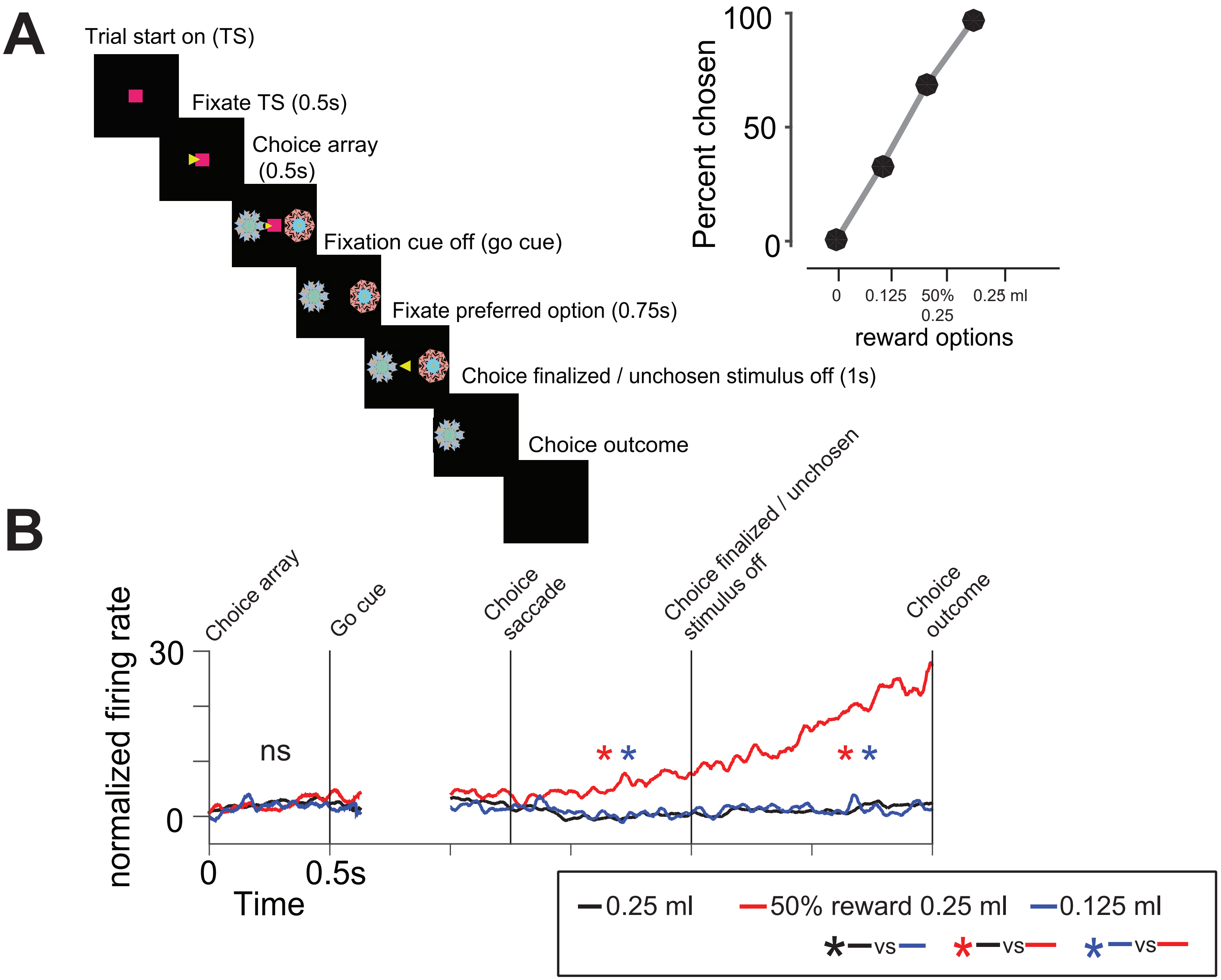

Supplementary Figure 7 – U+ neurons' responses during choice

(**A**) Choice-trial structure (left) and the monkeys choice behaviour (right). Monkeys made a choice between two CSs among the six well-learned CSs (three indicating reward amounts, and three indicating reward probabilities). The inset shows the proportion of trials the monkeys chose 0, 0.125, 50% 0.25, and 0.25 CSs over all other CSs. (**B**) Average normalized neuronal responses of U+ neurons during choice trials sorted by the monkeys' choices. Specifically, neuronal activity was sorted into trials in which the monkey chose the object associated with 0.25 ml of juice, (black), the object associated with 50% of 0.25 ml of juice (red), or 0.125 ml of juice (blue). Asterisks above task epochs indicate statistical differences amongst the 3 trial types (first Kruskal Wallis test $p<0.01$ across three trial types, then Wilcoxon rank sum tests; $p<0.01$; inset shows the meanings of asterisk color). Normalization was done by subtracting the average activity during trial start fixation epoch.

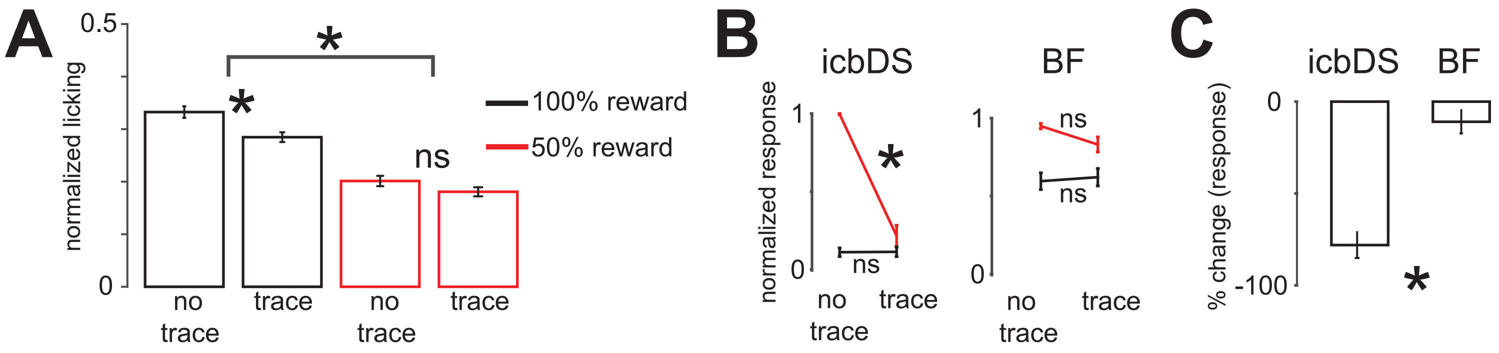

Supplementary Figure 8 – Trace conditioning: behaviour and comparison of striatum (icbDS) and basal forebrain U+ neurons' responses

(**A**) Licking behavior of two monkeys during trace conditioning (measured from CS presentation to outcome). The licking behavior during 50% reward trace and non-trace trials was not significantly different ($p>0.05$). Asterisks indicate significant differences ($p<0.05$; Wilcoxon rank sum test). Error bars denote standard error. (**B**) Comparison between ramping uncertainty selective neurons in the icbDS and the medial basal forebrain (BF). Average normalized responses of icbDS U+ neurons (left; $n = 32$) and BF uncertainty selective neurons (right; $n=17$) during trace and no-trace conditions. Responses are shown for 100% (black) and 50% (red) CSs separately. Asterisk denotes significant change (sign rank test; $p<0.05$). (**C**) Uncertainty response % change (trace versus no-trace) for icbDS and BF. Asterisk denotes significant difference (Wilcoxon rank sum test; $p<0.05$).

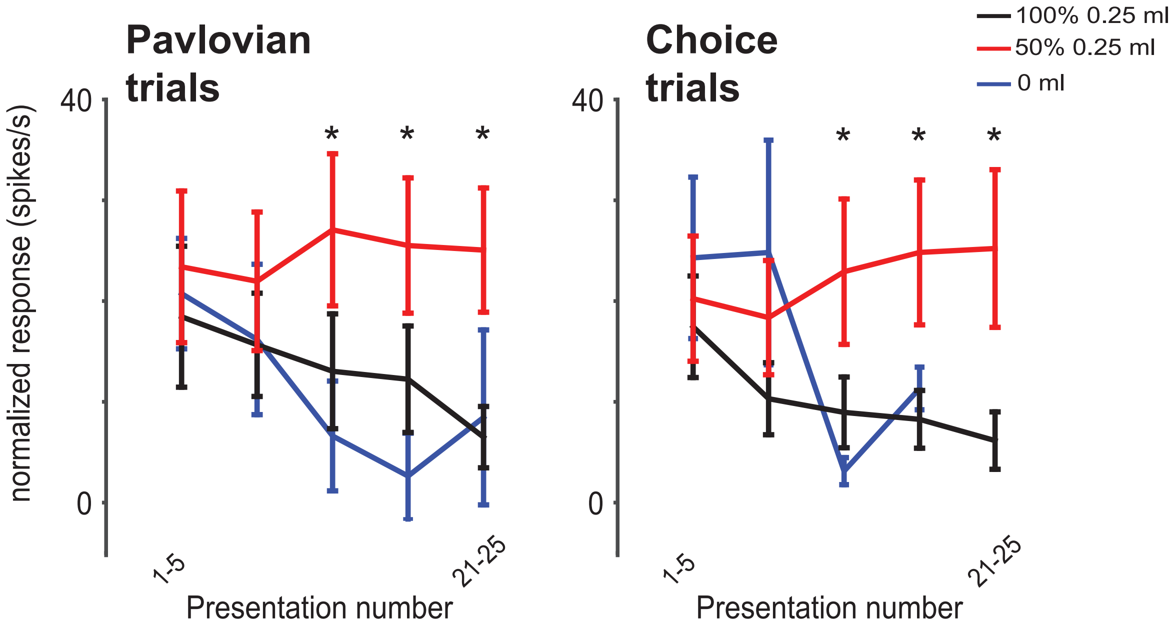

Supplementary Figure 9 – U+ neurons' respones during learning for Pavlovian and choice trials.

Binned neuronal population response across learning (30 learning sessions) shown separately for 100, 50, and 0% reward associated novel objects. Asterisks indicate significant variance across the three conditions ($p<0.05$; Kruskal-Wallis test).

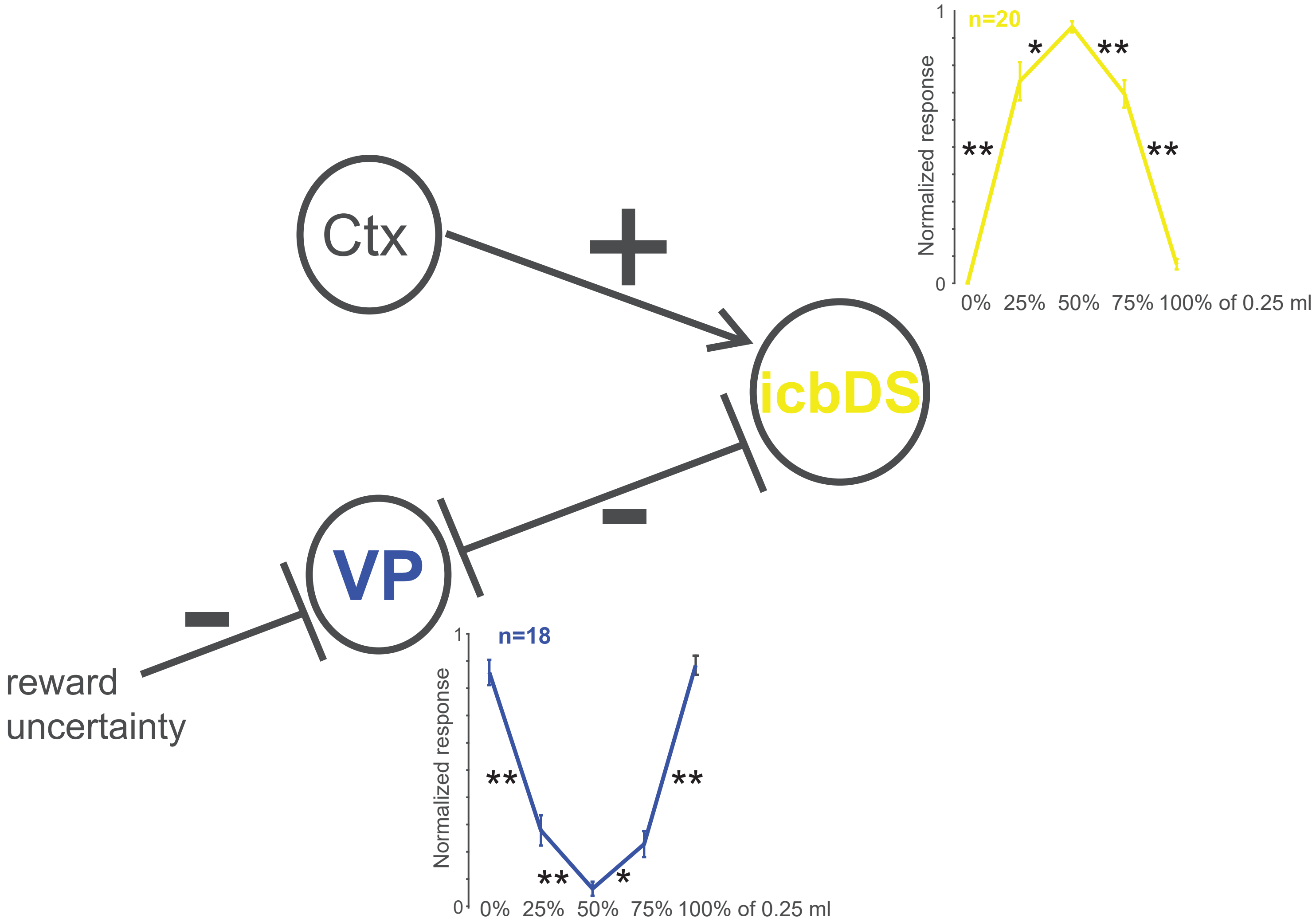

Supplementary Figure 10 – The ventral pallidum as one source of uncertainty signals in the striatum

The ventral pallidal regions are known to send inhibitory projections to icbDS. These regions contain uncertainty-suppressed neurons (Ledbetter, Chen, Monosov, 2016; Journal of Neuroscience). Here, we present a schematic of a hypothetical gating of icbDS by pallidal inputs. When the pallidum is inhibited by uncertainty, cortical inputs can further shape and drive the icbDS response.

We recorded the activity of 23 uncertainty-sensitive neurons in the ventral pallidum while two monkeys (B and W) experienced familiar/over-trained CSs associated with 0, 25, 50, 75, and 100 % chance of reward delivery (same as in Experiment 2). 18 neurons were suppressed by uncertainty, 5 were enhanced. Consistent with the proposed circuit, we found that the average CS responses of the 18 VP uncertainty-suppressed neurons (blue) form approximately an inverted mirror image of the response function of icbDS U+ neurons (shown in yellow). ** - $p < 0.01$ (sign rank test); * - $p<0.025$ (sign rank test).
